# An Assessment of the Effects of Guanidinoacetic Acid on the Performance and Immune Response of Laying Hens Fed Diets with Three Levels of Metabolizable Energy

**DOI:** 10.3390/ani14111675

**Published:** 2024-06-04

**Authors:** Santiago García-Gómora, Gabriela Gómez-Verduzco, Claudia C. Márquez-Mota, Arturo Cortés-Cuevas, Oscar Vicente Vazquez-Mendoza, Ernesto Ávila-González

**Affiliations:** 1 Departamento de Medicina y Zootecnia de Aves, Facultad de Medicina Veterinaria y Zootecnia, Universidad Nacional Autónoma de México, Avenida Universidad 3000, Ciudad de México 04510, Mexico; s.w.garcia168@gmail.com; 2 Departamento de Nutrición Animal y Bioquímica, Facultad de Medicina Veterinaria y Zootecnia, Universidad Nacional Autónoma de México, Avenida Universidad 3000, Ciudad de México 04510, Mexico; 3 Centro de Enseñanza, Investigación y Extensión en Producción Avícola CEIEPAv, Tláhuac, Ciudad de México 13300, Mexico; cuevasarturo@yahoo.com (A.C.-C.); avilaernesto@yahoo.com (E.Á.-G.); 4 Facultad de Medicina Veterinaria y Zootecnia, Universidad Autónoma del Estado de México, Toluca 50295, Mexico; osvam2009@gmail.com

**Keywords:** guanidinoacetic acid, laying hens, low energy levels

## Abstract

**Simple Summary:**

Annually, the demand for food to supply the world’s population is increasing, and the demand for poultry products is constantly increasing. Therefore, to meet these requirements, it is necessary to implement low-cost poultry diets. One way to reduce the cost of production is with the use of feed additives that increase the amount of metabolizable energy, such as guanidinoacetic acid (GAA). In the present study, the effect of GAA in diets with three levels of metabolizable energy (ME) was evaluated. The addition of GAA to low-ME diets maintained egg production and egg mass at similar levels to those of hens fed a high-ME diet, which indicates that adding GAA is a promising nutritional strategy for reducing the level of ME in the diets of laying hens.

**Abstract:**

Different levels of metabolizable energy (ME) and the inclusion of guanidinoacetic acid (GAA) in the diet of 53-week-old Lohmann LSL-CLASSIC hens were used to evaluate its effect on reproductive parameters, egg quality, intestinal morphology, and the immune response. Six diets were used in a 3 × 2 factorial design, with three levels of ME (2850, 2800, and 2750 kcal/kg), and with (0.08%) or without the inclusion of GAA. The addition of GAA to diets with low levels of ME increased (*p* < 0.05) egg production and egg mass. Moreover, hens fed with 2800 kcal/g without GAA had the highest concentration (*p* < 0.05) of serum interleukin IL-2, while those fed diets with the same amount of ME but supplemented with 0.08% GAA had the lowest concentration. Finally, the inclusion of 0.08% GAA increased (*p* < 0.05) the concentration of vascular endothelial growth factor (VEGF), regardless of the ME level in the diet. This study highlights the potential role of GAA in decreasing the energy level of ME (50–100 kcal/g) in the feeding of hens and in the modulation of specific immune responses. Further research is recommended to fully understand the mechanisms of action of GAA on the mechanism target of rapamycin and its relationship with the immune response.

## 1. Introduction

In Mexico, poultry production is an economic activity of great importance. Chicken production contributes 15.2% of the total livestock production of the country, with a per capita consumption of 35.3 kg of chicken meat. Moreover, egg production represents 12.6% of the national livestock production, with a per capita consumption of 24 kg [1]. Given the growing demand for poultry products, the use of food additives that favor muscle growth and the development of birds, including secondary metabolites, probiotics, prebiotics, and antimicrobial peptides (AMPs), among others, has increased [2]. Among these alternatives is guanidinoacetic acid (GAA), the natural precursor of creatine (Cr), which is synthesized from arginine and glycine in organs such as the kidneys, pancreas, liver, endocrine tissues, and skin [3,4]. The main function of GAA is to optimize energy use, which is achieved through the efficient conversion of Cr. Therefore, the inclusion of GAA in the diet causes an increase in Cr levels in the muscles, which stimulates muscle metabolism by increasing the levels of phosphocreatine (PCr) and adenosine triphosphate (ATP) [5].

Recent research has shown that GAA, as a feed additive, has considerable potential to improve production performance, thereby improving the quality of beef [6] and poultry [3]. The catabolism of GAA occurs in the small intestine via the action of pancreatic enzymes such as trypsin and elastase in conjunction with dipeptidases produced at the border of the intestinal barrier, releasing arginine and glycine. These amino acids are transported through the intestinal apical membrane [7,8] and participate in different aspects of metabolism [9,10].

It has been reported that arginine and glycine are essential for activating the mammalian target of the rapamycin (mTOR) signaling pathway, which regulates different biological processes, such as growth control, cell proliferation, protein synthesis, and metabolic processes. Besides the activation of the mTOR signaling pathway by arginine and glycine, it has been reported that GAA, through the increase in Cr, enhances skeletal muscle development and the regulation of different genes, such as myogenic regulatory factors (MRFs) and myocyte enhancer factor 2 [6]. Administering 1.2 g/kg of GAA in the diet of commercial birds increases productivity parameters [3], and under caloric stress, the joint administration of GAA and Cr increases these parameters without the need to add arginine to the diet [11]. Meanwhile, in broilers, supplementation with 0.5 g/kg of GAA increases the size, width, and absorption surface area of the intestinal villi in the duodenum, jejunum, and ileum [12]. In a study carried out in hens in the reproductive stage, supplementation with 1.5 g/kg of GAA increased daily egg production and strengthened the hepatic antioxidant response [13]. However, in that study, the effect of GAA on the intestinal characteristics of the animals was not evaluated.

In addition to the use of feed additives, metabolizable energy (ME) is a crucial factor in poultry production, and it has been reported that energy levels of 2850 kcal/kg of ME promote productivity in birds [14]. However, reaching these levels represents a high cost for producers, which is why strategies that combine new technologies, and the formulation of diets, are currently being evaluated to reduce them. For example, in prestart-stage broilers, a combination of diets with 2850 kcal/kg of ME and 1.2 g of GAA/kg compensates for the energy deficiencies of a low-energy diet [15]. This indicates that the combination of these factors may be beneficial for production; however, most studies evaluating the effects of different levels of ME and the use of GAA have mainly focused on broilers. Meanwhile, there is scarce information about the relationship between GAA and the immune response, for instance, in a broiler study under heat stress, it was demonstrated that supplementation with 0.6% GAA does not improve the immune response [16]. As previously stated, GAA catabolism generates arginine and glycine, which activates the mTOR signaling pathway, and it has been reported that this activation might regulate the expression of anti- and pro-inflammatory interleukins and cytokines [17]. In mammals, it has been reported that the mTOR signaling pathway may be key in the activation and inhibition of certain mechanisms of the immune response, which can result in health or disease, for instance, the levels of cytokine and interleukin expression. It is interesting to note that factors such as oxygen levels, nutrient levels, and energy levels can trigger either an inhibitory or activating effect in the corresponding phosphorylation cascades [18]. For this reason, in the present study, we evaluated the possible effect of GAA on the immune response of hens to further understand its beneficial effect on poultry health. Therefore, the use of GAA with decreased energy levels might improve the productive performance, egg quality, and intestinal health of hens.

## 2. Materials and Methods

This research was carried out at the CEIEPAV facilities located in Manuel M. López s/n, Santa Ana Poniente, Tláhuac, 13300 CDMX, Mexico.

### 2.1. Animal and Diet Management

All the procedures and handling of the birds were previously approved by the Institutional Subcommittee for the Care and Use of Experimental Animals (SICUA-FMVZ-UNAM) with the number SICUAE (MC-2022/3-3).

A total of 576 53-week-old laying hens of the Lohmann LSL-CLASSIC lineage were randomly allocated to six groups with different treatments (*n* = 8 replicates/treatment; 12 hens/group). The birds were housed in California-type cages (3 birds per cage, with dimensions of 40 × 45 × 45 cm). The animals were distributed into six groups with different experimental treatments: Treatment 1 (T1), a diet with 2850 kcal/kg of ME; Treatment 2 (T2), a diet with 2800 kcal/kg of ME; Treatment 3 (T3), a diet with 2750 kcal/kg of ME; Treatment 4 (T4), a diet with 2850 kcal/kg of ME + 0.08% GAA (GuanAMINO^®^ Evonik Industries México SA de CV, Ciudad de México, Mexico); Treatment 5 (T5), a diet with 2800 kcal/kg of ME + 0.08% GAA (GuanAMINO^®^ Evonik Industries México SA de CV, Ciudad de México, Mexico); and Treatment 6 (T6), a diet with 2750 kcal/kg of ME + 0.08% GAA (GuanAMINO^®^ Evonik Industries México SA de CV, Ciudad de México, Mexico). The diets were balanced according to the nutritional recommendations for the Lohmann LS-Classic strain [19] and had a corn–soy base (Table 1).

### 2.2. Productivity Variables

Over 12 weeks, the consumption of feed/bird/day (g), percentage of hens/day (%), egg mass/bird/day (g), and feed conversion (kg–kg) were recorded. Egg mass was calculated as the product of the egg weight and the percentage of laying hens divided by 100. All variables were corrected according to mortality.

### 2.3. Egg Quality

The egg quality parameters were evaluated at 0, 4, 8, and 12 weeks of experimentation. Eighty eggs were collected per treatment (five eggs per replicate, with eight replicates), and the variables of egg weight, albumin levels, and Haugh units were evaluated using a TSS^®^ QCD system (Technical Services and Supplies Ltd., Chessingham Park Dunnington YORK YO19 5SE United Kingdom). The color of the yolk was determined by means of a colorimetric instrument, and the thickness and resistance to fracture of the shell were also evaluated.

### 2.4. Intestinal Histology

The length, villus width, crypt depth, and villus depth-to-crypt ratio (RVC) of the duodenum and jejunum were evaluated. Sections of approximately 2 cm were obtained from the middle third of the duodenal loop, as well as from the jejunum, 5 cm before the Meckel diverticulum. Intestinal lavage was performed prior to depositing the samples in 10% formalin (pH 7.2), and the samples were stored until analysis. The samples were processed and embedded in paraffin for staining using hematoxylin and eosin [20]. The histological sections were evaluated at a 40× magnification for both the height and width of the villi and the depth of the crypts using an optical microscope (Leica DM750^®^, Wetzlar, Germany) coupled to a camera for digital microscopy. The representative fields were photographed, and digital images were captured for morphometric analysis. The LAS EZ Leica software v3.4 was used for the morphometric measurements of height, villus width, and crypt depth. For the height of the villi, a measurement was taken from the apex to the lamina propria. The width of the villi was measured as the distance from the junction of one villus to the other to the basement membrane of the epithelial cell in the lower third of the length of the villi (the width of the base of the villi). The crypt depth was measured as the distance from the junction to the basement membrane of the epithelial cells at the bottom of the crypt, as previously reported in [21].

### 2.5. Determination of Interleukin and Cytokine Levels in Serum

Peripheral blood was aseptically collected from 42 hens (7 animals per treatment). The sera were separated and stored at −20 °C until analysis. In the present study, the Merck Miliplex Cytokine Assay Kit (Cat GCYT1-16K. Millipore Corporation Merck, Darmstadt, Germany) was used according to the supplier’s instructions [22].

### 2.6. Statistical Analysis

This experiment had a 3 × 2 factorial arrangement, where 1 factor was the 3 levels of ME (2850, 2800, and 2750 kcal), and the other factor was the inclusion of GAA (0 and 0.08%). The data obtained were analyzed with RStudio (RStudio 2024.04.1+748) [RStudio Team, Boston, MA, USA, 2020]. First, the assumptions of statistical independence, homogeneity of variance, and a normal distribution of the data were corroborated. Subsequently, the means of the parameters (*p* > 0.05) were compared via Tukey analysis using the Agricolae library and a linear model routine (lm).

## 3. Results

### 3.1. Productivity Variables

No differences in egg weight were detected between the groups, as shown in Table 2. The egg mass decreased significantly (*p* < 0.05) when the ME level decreased. In addition, an interaction effect between the ME and GAA was detected (*p* < 0.05), and the egg mass increased with the addition of GAA in the low-ME diet. A significant effect of the ME levels was also observed, whereby the animals fed diets with high ME levels consumed less food (*p* < 0.05) than those whose diet had lower ME levels. However, no significant effect of GAA in the diet on feed consumption was observed. Regarding the interactions, the birds that consumed 2800 kcal and GAA had the lowest feed consumption compared with the other experimental groups. In terms of the percentage of laying, there was a significant decrease (*p* < 0.05) in the percentage of animals fed low-energy diets (2750 kcal); however, when GAA was included in these diets, the percentage of laying increased. Meanwhile, the feed conversion was more efficient in the hens that consumed diets with high ME levels.

### 3.2. Egg Quality

The Haugh units, the resistance, and the thickness of the shell, and the color of the yolk, did not significantly change in response to the treatments (Table 3).

### 3.3. Intestinal Histology

The level of ME in the diets had no effect on the height or width of the villi (Table 4, Figure S1) or on the depth of the crypts in the duodenum. However, differences were observed in the animals fed 0.08% GAA, which presented a lower villi height (1974.03 µm) but a greater width (272.99 µm) and greater depth of crypts (348.02 µm) than did the animals that did not consume GAA. In the jejunum (Table 4, Figure S2), the dietary ME level did not affect the height of the villi. However, diets with a greater ME level increased the width of the villi and the depth of the crypts. With respect to the inclusion of GAA, the animals fed diets supplemented with 0.08% GAA presented the smallest villi width compared with those that did not consume GAA. Regarding the interaction between the energy level and GAA concentration, only the depth of the crypts in the jejunum was affected. Animals fed a high-energy diet (2800 kcal) supplemented with 0.08% GAA had the greatest crypt depth (Table 4).

### 3.4. Determination of Interleukin and Cytokine Levels in Serum

The inclusion of 0.08% GAA resulted in a significant decrease in the levels of several cytokines (Table 5): IFN-γ (347.1 pg/mL), IL-16 (115.2 pg/mL), IL-2 (463.5 pg/mL), IL-6 (337.8 pg/mL), IFNα (248.1 pg/mL), M-CSF (1156.3 pg/mL), MIP-1b (111.5 pg/mL), and MIP-3a (140.6 pg/mL). In addition, an increase in the VEGF concentration (3297.0 pg/mL) was observed in the animals that consumed GAA compared with that in the animals that did not consume GAA. In relation to the interaction between the contribution of GAA and the ME levels in the diet, the animals fed the diet with the highest ME level (2800 kcal) without the addition of GAA presented the highest concentration of IL-2 (889.9 pg/mL). Conversely, those who consumed the same diet but with the inclusion of 0.08% GAA showed the lowest concentration of this interleukin (372.3 pg/mL). The inclusion of 0.08% GAA increased the concentration of VEGF, regardless of the energy level in the diet.

## 4. Discussion

GAA, a stable and highly available additive, has been reported to optimize energy use by efficiently converting Cr in the bodies of birds, cattle, and humans [4,23,24]. It has been reported that GAA stimulates the insulin response and the secretion of insulin-like growth factor-1 (IGF1) [24], and, as previously stated, GAA catabolism generates arginine and glycine, and, all together, hormonal signals and amino acid levels stimulate the activity of the mTORC1 signaling pathway [25]. For instance, in pigs, it has been reported that 1.2 g/kg of GAA increases the mRNA expression of ribosomal protein S6 kinase beta-2 (*RPS6KB2*) and the protein abundance of eukaryotic translation initiation factor 4E (eIF4E)-binding protein 1 (4E-BP1), which are gene and protein targets, respectively, of the mTORC signaling pathway [26]. However, its use in laying hens has shown mixed results [13,27,28,29]. This is attributable to the lack of consensus on the optimal levels of inclusion of this additive. The addition of 1.0 to 1.5 g/kg of GAA improved the laying of hens in [13]. Likewise, doses of 0.6 and 1.2 kg of GAA improved feed conversion in [28], while levels of 0.057% to 0.171% of GAA did not modify the productive characteristics of Hy-Line W-36 hens in [29].

In this study, the inclusion of 0.08% GAA in the feed of laying hens did not alter feed conversion, a result that contrasts with previous reports that suggested an increase with the inclusion of 0.06% GAA in chickens [30]. This discrepancy could be attributed to differences in the energy requirements and the productive characteristics of each species. In laying hens, feed conversion is strongly influenced by egg production cycles [31]. 

Additionally, egg production was not affected by the inclusion of 0.08% GAA. Importantly, GAA is derived from the combination of arginine and glycine. Arginine has been shown to increase egg production by increasing the secretion of luteinizing hormone. In previous studies, the inclusion of 1.02% arginine in the diet increased egg production in chickens [32], while in geese, an arginine concentration of 0.1% increased egg production [33].

Therefore, the GAA concentration used in this study was not sufficient to observe significant changes in the productivity parameters of laying hens. However, it is important to highlight that supplementation with 0.08% GAA maintained production in the hens fed a diet with a caloric deficit of 50 kcal. Further studies with higher inclusion levels are necessary to evaluate its possible beneficial effect when included in low-calorie diets. 

An increase in egg production and mass was observed in diets with low ME levels that included 0.08% GAA. This result partially coincides with that reported by some authors, who indicated that GAA increases Cr synthesis and improves reproductive behavior in hens [23]. However, the results of the present study contrast with the findings of other studies that indicate that the inclusion of 0.46% GAA reduces egg mass [34]; however, its inclusion in the range of 0.057% to 0.171% has no effect [29].

A crucial factor in egg production is the characteristics of the shell. It has been reported that the inclusion of 0.6 g/kg of GAA in the diet increases the thickness and resistance of the shell, although it does not affect the Haugh units or the color of the yolk [33]. In our study, no significant effects on the Haugh units, shell strength, shell thickness, or yolk color were observed due to the ME level or the inclusion of 0.08% GAA. This suggests that although GAA can influence the characteristics of the egg and the shell, it is necessary to increase the inclusion dose to observe these effects.

The morphological characteristics of the duodenum and jejunum are modified by dietary ME levels. It has been reported that diets with high ME levels increase the height of the villi in the duodenum [35]. In the present study, the ME level did not modify the morphological characteristics of the duodenum. However, GAA supplementation increased the width of the villi and the depth of the crypts and decreased the height of the villi, which is contrary to what was reported by Peng et al. in 2003, in which an improvement in intestinal morphology was observed in broilers subjected to heat stress supplemented with 0.6 g/kg of GAA [36]. It has been shown that the length of the villi improves the absorption capacity of nutrients in the intestine and that deep crypts indicate high metabolic activity in the tissue, so modifications in these characteristics may indicate a reduction in the absorption of nutrients [37,38,39]. Moreover, it has been reported that the absorption capacity can be an indicator of the health of birds. In particular, a greater intestinal absorption surface area can be indicative of a probable inflammatory process [40].

In the case of the production of interleukins, the addition of GAA decreased the concentrations of IL-2 and IFNγ. These results are contrary to those reported by Li et al. in 2023, wherein the addition of 0.6 g/kg of GAA caused an increase in the concentrations of IL-2 and IFNγ in laying hens exposed to heat stress. The transcription of interleukin, cytokine, and chemokine genes involves the expression of immediate genes, the early activation genes, and the late activation genes, and this may be a determining factor in the results of this research compared with what was previously reported by Li et al. in 2023 [41]. In addition, the activation of genes encoding these mediators of the immune response can be influenced by various factors, among which various regulatory sequences within these genes stand out, resulting in positive or negative regulation [42]. IL-2 has an important role in the generation and maintenance of immune tolerance [43]. Meanwhile, IFNγ is essential in protection against infections [44]. Therefore, we can hypothesize that the inclusion of GAA in hens’ feed might contribute to immune regulation and stimulate health benefits.

The results of this study suggest that GAA supplementation in the diet of hens during production may have a significant impact on the immune response. The inclusion of GAA in the diet resulted in a reduction in food intake and caused intestinal morphological changes, such as a reduction in the height of the villi and an increase in the width and depth of the crypts of certain segments of the intestine. These changes suggest an adaptation of the intestine to improve the absorption of nutrients and indicate a possible beneficial effect on the intestinal health of birds. Similarly, the addition of GAA decreased the levels of various proinflammatory cytokines and increased the level of VEGF, suggesting that GAA may have anti-inflammatory and angiogenesis-promoting effects. In human models, it has been suggested that the addition of GAA inhibits the activity of the mTORC1 signaling pathway, causing a decrease in the release of various interleukins and cytokines, with vascular endothelial growth factor (VEGF) being the only one that increases because of GAA [45]. However, in poultry, the mTORC1 signaling pathway and related phosphorylation mechanisms have not yet been investigated. Thus, it is important to elucidate whether these mechanisms are homologous to those reported in mammals. The interaction between the ME level in the diet and the inclusion of GAA was also relevant, showing that diets with a high energy content plus GAA achieved better feed conversion and clear alterations at the morphological level of the intestine, together with changes in cytokine levels. These findings confirm the importance of including GAA in the formulation of diets aimed at improving the intestinal health and production of poultry.

## 5. Conclusions

The present study showed that the inclusion of 0.08% GAA in diets with lower ME levels (50 to 100 kcal/kg) increases egg production and egg mass, as it optimizes energy use through efficient creatine conversion. The observed changes in gut morphology and cytokine levels suggest that GAA could influence gut health and hens’ immune responses, promoting greater nutrient absorption and, potentially, a reduced inflammatory state. These effects are promising but still require further exploration to fully understand the underlying mechanisms and practical implications of GAA supplementation in poultry diets. Future research should consider a longitudinal evaluation of the effects of GAA on production and bird health throughout different production cycles to provide more detailed and practical guidance for poultry producers.

## Figures and Tables

**Table 1:** Composition of experimental laying hens’ diets.

Ingredients (%)	T1	T2	T3	T4	T5	T6
Metabolizable energy	2850 kcal/kg	2800 kcal/Kg	2750 kcal/kg	2850 kcal/kg	2800 kcal/kg	2750 kcal/kg
Yellow corn	57.2	58.48	59.76	57.2	58.48	59.76
Soybean meal	23.86	23.68	23.49	23.86	23.68	23.49
Calcium carbonate	10.19	10.19	10.2	10.19	10.19	10.2
Wheat bran	3	3	3	3	3	3
Vegetable oil	3.43	2.33	1.24	3.43	2.33	1.24
Orthophosphate	0.94	0.93	0.93	0.94	0.93	0.93
Salt	0.4	0.4	0.4	0.4	0.4	0.4
Min/vit. premix CEIEPAv layer *	0.25	0.25	0.25	0.25	0.25	0.25
99% L-methionine	0.25	0.25	0.24	0.25	0.25	0.24
L-lysine-HCl	0.12	0.12	0.12	0.12	0.12	0.12
FREE-TOX	0.1	0.1	0.1	0.1	0.1	0.1
Yellow pigment	0.1	0.1	0.1	0.1	0.1	0.1
L-threonine	0.05	0.05	0.05	0.05	0.05	0.05
Larvadex	0.05	0.05	0.05	0.05	0.05	0.05
Bacitracin	0.03	0.03	0.03	0.03	0.03	0.03
Antioxidant ^•^	0.015	0.015	0.015	0.015	0.015	0.015
Phytase	0.01	0.01	0.01	0.01	0.01	0.01
Canthaxanthin	0.002	0.002	0.002	0.002	0.002	0.002
Total	100	100	100	100	100	100
GAA ^&^				0.08	0.08	0.08

* Mineral and vitamin premix provided: 12,000,000 IU of vitamin A, 2,500,000 IUP of vitamin D3, 15,000 IU of vitamin E, 2000 mg/kg of vitamin K3, 2250 mg/kg of vitamin B1, 7500 mg/kg of vitamin B2, 45,000 mg/kg of vitamin B3, 12,500 mg/kg of vitamin B5, 3500 mg/kg of vitamin B6, 20 mg/kg of vitamin B12, 1500 mg/kg of folic acid, 125 mg/kg of biotin, 300 mg/kg of iodine, 200 mg/kg of selenium, 200 mg/kg of cobalt, 50,000 mg/kg of iron, 12,000 mg/kg of copper, 50,000 mg/kg of zinc, and 110,000 mg/kg of manganese. ^&^ On top; ^•^ BHT.

**Table 2:** Productive performance in Lohmann LSL-CLASSIC laying hens (53–65 weeks of age) fed different energy sources with or without GAA.

Metabolizable Energy (kcal/kg)	GAA (%)	Egg Production (%)	Egg Weight (g)	Egg Mass (g)	Feed Consumption (g)	Feed Conversion Rate
2850	-	95.3 ^a^	63.5	60.5 ^a^	111.0 ^b^	1.8 ^b^
2800	-	93.8 ^ab^	63	59.1 ^b^	111.1 ^ab^	1.9 ^ab^
2750	-	93.3 ^b^	63.3	59.3 ^ab^	113.6 ^a^	1.9 ^b^
*p*-value		0.019	0.267	0.023	0.049	0.002
-	0	93.3	63.2	59.5	112.4 ^a^	1.9
-	0.08	94.3	63.4	59.8	111.4 ^b^	1.9
*p*-value		0.414	0.499	0.496	0.278	0.115
2850	0	95.2 ^a^	63.2	60.2 ^ab^	110.2 ^ab^	1.8
2800	0	94.4 ^ab^	63.3	59.8 ^ab^	113.6 ^a^	1.9
2750	0	92.0 ^b^	63.2	58.6 ^b^	113.5 ^a^	1.9
2850	0.08	95.3 ^a^	63.9	60.9 ^a^	109.5 ^ab^	1.8
2800	0.08	93.2 ^ab^	62.8	58.5 ^b^	108.7 ^b^	1.9
2750	0.08	94.6 ^ab^	63.5	60.1 ^ab^	113.7 ^a^	1.9
*p*-value		0.026	0.105	0.03	0.022	0.414
SEM		0.318	0.123	0.237	0.506	0.01

The absence of literals between the means of each column indicates that there were no significant statistical differences (a > b) (*p* < 0.05). SEM: standard error of the mean.

**Table 3:** Egg quality in Lohmann LSL-CLASSIC laying hens (53–65 weeks of age) fed different energy sources with or without GAA.

Metabolizable Energy (kcal/kg)	GAA (%)	Haugh Units (HUs)	Egg Shell Resistance (Kgf/cm^2^)	Egg Shell Thickness (μm)	Yolk Color FAN DSM
2850	-	93.7	4.5	349	11.4
2800	-	93.6	4.5	348	11.2
2750	-	92.9	4.6	350	11.8
*p*-value		0.4	0.4	0.1	0.6
-	0	93.4	4.5	348	11.6
-	0.08	93.5	4.5	350	11.3
*p*-value		0.781	0.973	0.811	0.665
2850	0	93.9	4.6	355	11.4
2800	0	93.9	4.4	345	11.1
2750	0	92.3	4.5	342	12.2
2850	0.08	93.6	4.4	343	11.3
2800	0.08	93.4	4.6	350	11.2
2750	0.08	93.6	4.6	357	11.4
*p*-value		0.386	0.022	0.554	0.789
SEM		0.506	0.028	0.005	0.263

The absence of literals between the means of each column indicates that there were no significant statistical differences (*p* < 0.05). SEM: standard error of the mean.

**Table 4:** Intestinal structure of Lohmann LSL-CLASSIC laying hens (53–65 weeks of age) fed different energy sources with or without GAA.

Metabolizable Energy (kcal/kg)	GAA (%)	Duodenum			Jejunum		
		Villus Height (μm)	Villus Width (μm)	Crypt Depth (μm)	Villus Height (μm)	Villus Width (μm)	Crypt Depth (μm)
2850	-	2029.28	257.63	332.3	1336.47	207.81 ^a^	203.99 ^a^
2800	-	2015.8	259.63	310.76	1355.33	186.01 ^b^	209.55 ^a^
2750	-	2004.09	260.46	328.37	1302.01	200.66 ^ab^	178.50 ^b^
*p*-value		0.7278	0.9739	0.1973	0.2363	0.0249	0.0001
-	0	2058.75 ^a^	245.49 ^b^	299.59 ^b^	1345.05	205.10 ^a^	192.55
-	0.08	1974.03 ^b^	272.99 ^a^	348.02 ^a^	1317.49	191.23 ^b^	202.14
*p*-value		<0.001	0.008	<0.001	0.2885	0.0373	0.1244
2850	0	2061.89	239.68	306.79	1314.23	220.74	202.03 ^abc^
2800	0	2084.98	253.44	294.07	1413.71	184.44	191.26 ^bc^
2750	0	2029.38	243.36	297.93	1307.2	210.13	184.36 ^bc^
2850	0.08	2029.19	275.58	357.82	1358.7	194.9	205.95 ^ab^
2800	0.08	1969.71	265.82	327.45	1296.94	187.58	227.83 ^a^
2750	0.08	1923.2	277.57	358.8	1296.82	191.2	172.65 ^c^
*p*-value		0.0396	0.5859	0.5478	0.0376	0.1772	0.0061
SEM		31.601	12.673	12.675	31.703	8.098	7.607

The absence of literals between the means of each column indicates that there were no significant statistical differences (a > b > c) (*p* < 0.05). SEM: standard error of the mean.

**Table 5:** Assessment of immune response via the measurement of serum interleukin and chemokine levels and hemagglutination inhibition titers in Lohmann LSL-CLASSIC laying hens (53–65 weeks of age) fed different energy sources with or without GAA.

Metabolizable Energy (kcal/kg)	GAA (%)	IFNγ	IL-16	IL-6	IL-2	IFNα	M-CSF	MIP-1β	MIP-3α	VEGF
		(pg/mL)								
2850	-	942.4	246.8	848.3	704.3	703.8	3510	290	280.1	3062.7
2800	-	483	153.9	543.4	525.9	329.7	1647.2	167.7	166.4	2879.2
2750	-	662.8	191.9	721.3	549.3	492	2314.8	215.8	261.9	3099.3
*p*-value		0.46	0.56	0.69	0.22	0.44	0.45	0.49	0.4	0.4
-	0	1045.0 ^a^	280.0 ^a^	1070.8 ^a^	722.9 ^a^	769.0 ^a^	3825.1 ^a^	337.5 ^a^	331.7 ^a^	2730.5 ^b^
-	0.08	347.1 ^b^	115.2 ^b^	337.8 ^b^	463.5 ^b^	248.1 ^b^	1156.3 ^b^	111.5 ^b^	140.6 ^b^	3297.0 ^a^
*p*-value		0.02	0.02	0.01	0.01	0.03	0.03	0.01	0.01	0.001
2850	0	1463.2	367.8	1394.3	889.9 ^a^	1119.8	5698.5	468.3	407.5	2954.8 ^ab^
2800	0	861.7	251.6	940.2	679.5 ^ab^	607.3	3013	283	264.8	2418.1 ^b^
2750	0	810.2	220.5	878	599.3 ^ab^	579.8	2763.7	261.2	322.7	2818.5 ^ab^
2850	0.08	421.7	125.9	302.3	518.7 ^ab^	287.9	1321.5	111.8	152.6	3170.6 ^a^
2800	0.08	104.2	56.2	146.6	372.3 ^b^	52.1	281.4	52.4	67.9	3340.3 ^a^
2750	0.08	515.4	163.4	564.6	499.4 ^ab^	404.3	1865.9	170.4	201.1	3380.0 ^a^
*p*-value		0.54	0.16	0.12	0.03	0.46	0.16	0.06	0.11	0.002
SEM		146.54	33.55	136.65	45.5	112.23	568.37	40.21	37.29	81.38

The absence of literals between the means of each column indicates that there were no significant statistical differences (a > b) (*p* < 0.05). SEM: standard error of the mean.

## Data Availability

The datasets generated during and/or analyzed during this study are available from the corresponding author upon reasonable request.

## Supplementary Materials

The following supporting information can be downloaded at https://www.mdpi.com/article/10.3390/ani14111675/s1. Figure S1: Hematoxylin and eosin staining of duodenum; Figure S2: Hematoxylin and eosin staining of jejunum.
