# Supplementary material for: An Assessment of the Effects of Guanidinoacetic Acid on the Performance and Immune Response of Laying Hens Fed Diets with Three Levels of Metabolizable Energy
Source: Animals (Basel). 2024 Jun 4;14(11):1675. doi: 10.3390/ani14111675 (PMC11171344; doi:10.3390/ani14111675)
Supplement: Supplementary file 1 [file animals-14-01675-s001.zip › animals-3038793-supplementary.pdf]

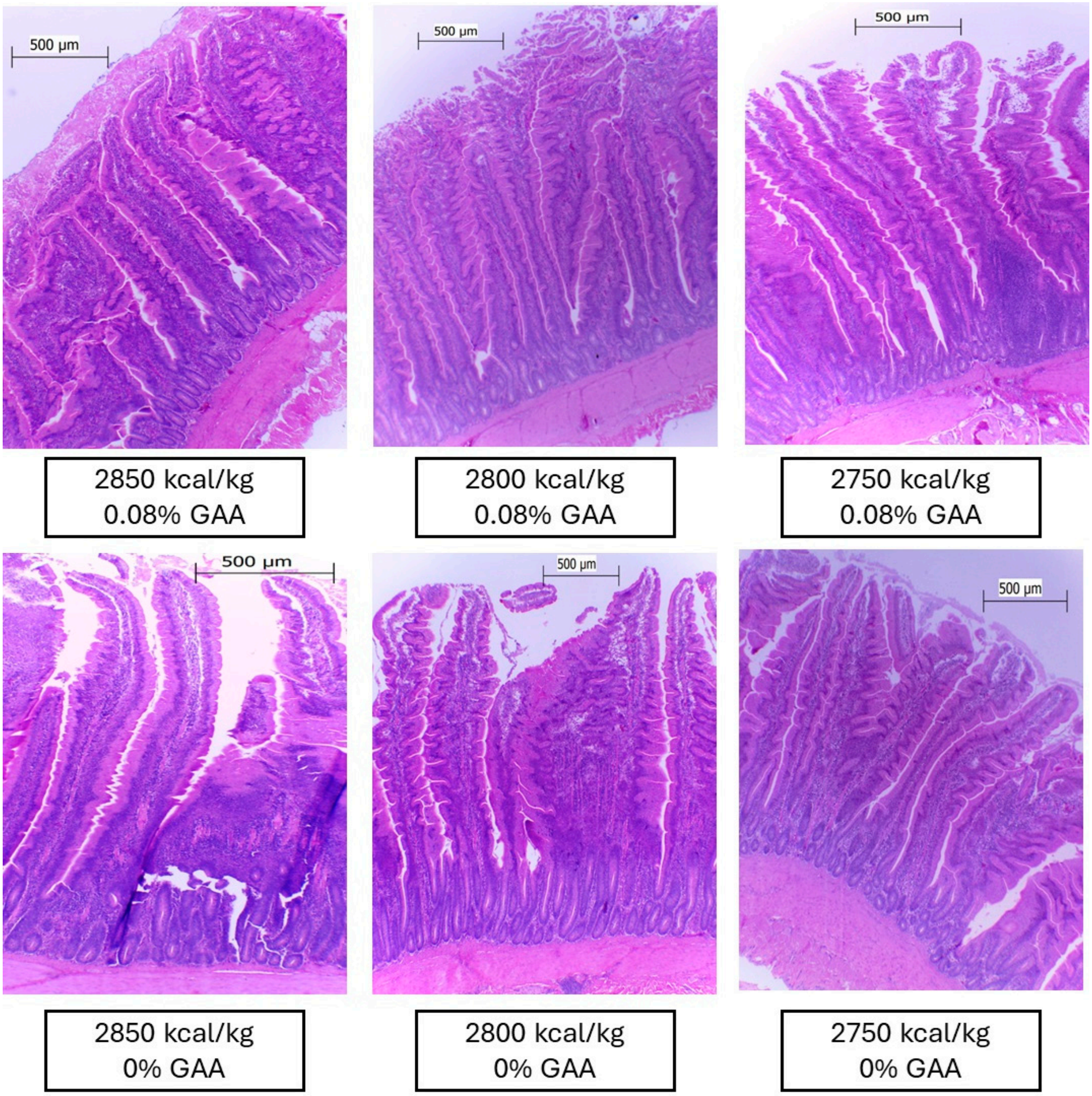

Figure S1. Hematoxylin and Eosin Staining of duodenum

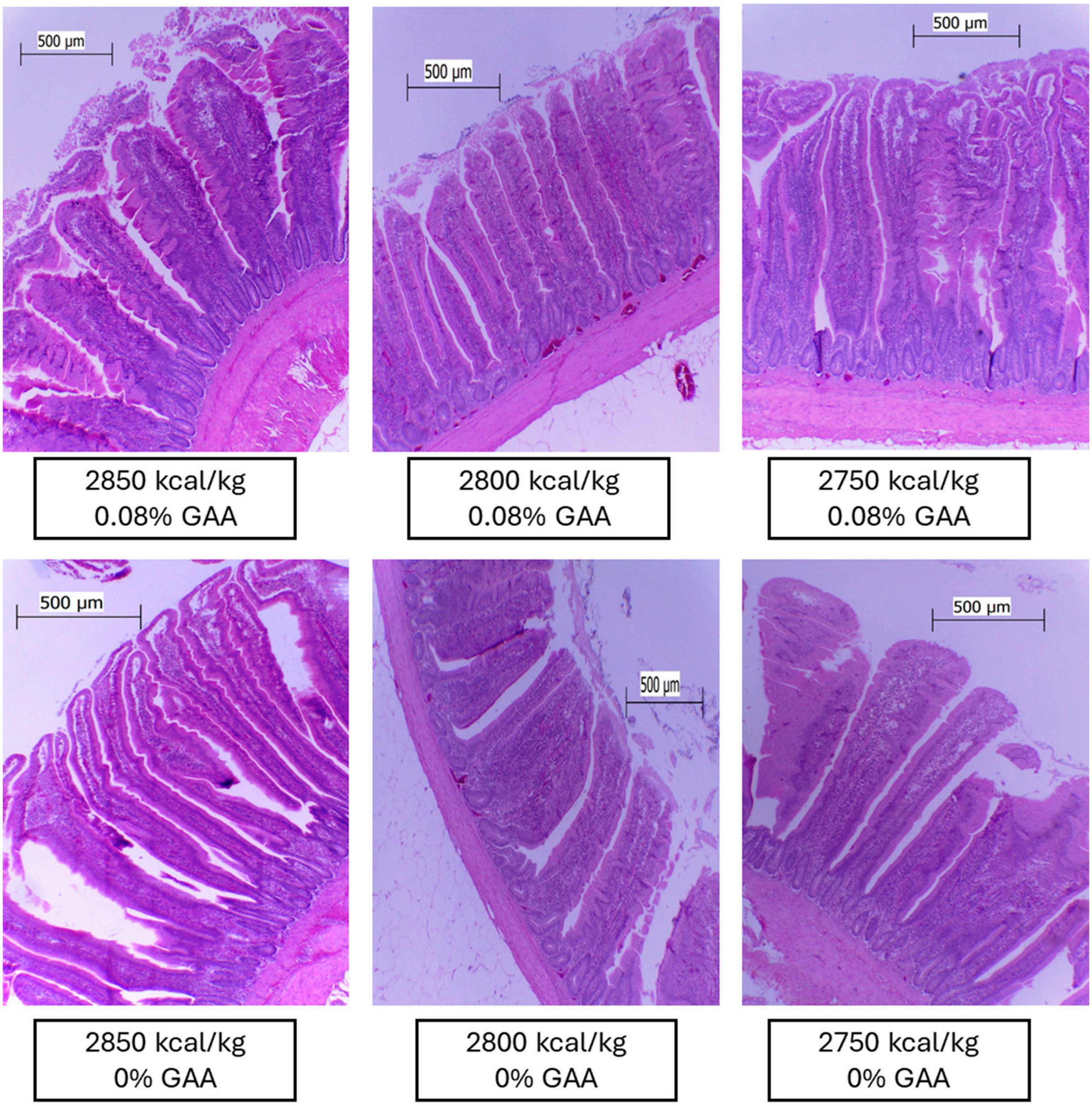

Figure S2. Hematoxylin and Eosin Staining of jejunum
